# Extraction of First Molars

**Published:** 1880-03

**Authors:** A. W. Harlan

**Affiliations:** Chicago


### EXTRACTION OF FIRST MOLARS.

BY A. W. HARLAN, OF CHICAGO.

The diversity of opinion concerning the treatment of these teeth is so great, and their proper treatment under all circumstances so difficult to determine on the first visit of the patient, that many operators either hurriedly extract them, or are satisfied to as hurriedly fill them with inferior materials, thus hastening their ultimate destruction. I desire, therefore, to offer a few words with reference to the proper time of extracting them, and a few indications therefor.

First.—In all mouths where it is apparant that both jaws alike give evidence of being contracted from a normal-shaped arch, and the teeth are large and imperfectly developed, my rule would be to extract all four of them at the same time, even though only one of them should be seriously affected by caries.

Second.—In all cases where the patient is presented previous to the eleventh year with one pulpless molar of the above class, and the other three already so badly decayed that one or more pulps are exposed, I would extract, even if the shape of the arch were normal, and no tendency existed to indicate that the teeth would be crowded or irregular in their final arrangement.

Third.—The rule is to extract when a patient is presented who has previously lost one of these teeth before the eleventh year, unless one of the bicuspids has also been extracted on the opposite side of the arch and not from the same maxilla. Then, perhaps it would be better to extract the corresponding bicuspid, but if the articulation of the teeth were interfered with by so operating to any appreciable degree, the bicuspid should be extracted, and the remaining molar from the maxilla from whence the other had been extracted. Further, I would never extract a molar, or the molars of the sixth year, for the purpose of correcting an irregularity of the teeth anterior to them, if they were all alive and of reasonably dense structure.

Fourth.—The rule would be to extract the sixth year molars after the eruption of the second bicuspids in those cases where the teeth are large and were erupted early, and are of a chalky structure, from a perfectly normal arch, as these teeth invariably show caries on their distal and mesial surfaces, while there are no teeth in contact with them, often as early as the seventh to the eighth year, and it is next to impossible to save them. They should be extracted about the tenth year.

Fifth.—It is proper to extract what are known as honey-combed teeth, and those teeth showing arrested development as a result of exanthematous diseases, and also those teeth which bear the marks of inherited disease—meaning by the above descriptions teeth almost wholly devoid of shape and destitute of enamel and already extensively decayed previous to the eighth or ninth year.

Conclusions.—My observations of the mouths of children and adults, and study of authorities, lead me to conclude that in the above-named cases, the prospect for the patient having a perfect and regular arch is many times greater than the most skillful treatment by filling ever possibly produced. In such cases there is no interruption of the eruptive process of the twelfth year molar, and at the period of the eruption of the dentes sapientiæ, they come far forward of what would have been the case in the event of the non-extraction of the sixth year molars. It must be understood that I do not advise the indiscriminate extraction of these teeth, you may see under what circumstances I do advocate their extraction.

This paper would be useless if the proper period for the extraction of the above teeth were not indicated. When we know that the molars were erupted at or about the sixth year, my rule is to extract not later than the tenth year; if they were erupted as late as the seventh year, I defer their extraction, if possible, until the eleventh year, and seldom later than that date.

One word more. It is claimed by some authorities that of late years a tendency has been observed by them indicating the total suppression of the third molars in the near future. Those who are fearful of that calamity will not view with favor the proposition to extract any of the above-named teeth, and they will undoubtedly continue to fill all such without reference to the future utility of a lesser number of teeth, or the ease and facility with which they may be cared for by patient and operator.

### DISCUSSION.

Dr. Crouse: The paper was too short in its descriptions to indicate the precise meaning of the essayist in regard to the times for extracting and the reasons for doing so. The paper advocates the removal of the remaining three if one has been lost. I do not understand why a calamity should be aggravated in that way. Doubtless there are some that can not be preserved; but it is only very seldom that I extract them. If an arch is very crowded, and can not be remedied in any other way, and the first molars were the poorest teeth in the mouth, would extract them. Some say that almost all first molars should be removed. I would reverse that, and say that almost all should be retained. There loss is one of the greatest injuries that children are constantly receiving. It happens partly by reason of the mistaken ideas of dentists as to their importance, and more because they are often decayed so early as to escape proper attention. The saving of them is one of the most difficult things we have to do, because they come to us in infancy. I sometimes fill the fissures with tin under water, as soon as they are erupted, so as to do it before they become sensitive.

Dr. K. B. Davis: Parents have a prevalent idea that these are temporary teeth, and bring them first when they are so far gone as to be aching. It is, therefore, our duty to instruct parents in regard to temporary and permanent teeth, and people should be encouraged to bring their children for the examination of their mouths. Would often fill these teeth with something else than gold.

Dr. Swain : I do not understand the paper as advocating at all the extermination of the sixth year molars ; but it gave some rules and directions in regard to the time and conditions for extracting them in the cases in which it is *necessary*. I know a case of a child who had one extracted three or four years ago, and now the median line has moved to one side. The persons interested dislike the condition very much. If *symmetry* is the most necessary condition, we would always extract both in the same jaw or none.

Dr. Black: So far in this discussion the special service which these teeth perform during the shedding of temporary teeth, has not been mentioned. They come up and antagonize about the time the removal of the temporary teeth begins, and they serve to keep the face and jaws in proper position during the breaking up and changing of the articulation of the teeth anterior to them during shedding and replacement. If one is lost the strain of the muscles will pull the jaw awry, and there are a great many such jaws. If a bicuspid is in place and articulating with its fellow, we may remove a molar, if necessary, which we would not otherwise. At this time of life, if both these teeth must come out eventually, they should be removed at the same time, and not several years apart, else the centre of the upper and under jaws will not correspond. We all prefer to save them, but there are cases in which it is impossible, though even in these we may often *hold on* to them till this special service during the eruption of the permanent teeth is accomplished.

Dr. Cushing: The remarks of Dr. Black in regard to the importance of keeping these teeth for the purpose mentioned, have great weight. The mesial line will often move to one side, nearly or quite the width of a tooth. I do not understand the paper to advocate extraction, unless reasonably sure that they must be lost. There are many cases in which they certainly have to be extracted in the end, though it may be best to fill them for awhile. We should regard the future conditions likely to arise, and do the best we can for the future years. What will be likely to give our patient the most useful mouth for the longest time.

Dr. Swain: The sixth year molars, when first erupted, meet face to face, instead of the upper ones covering over the anterior two-thirds of the under ones, as in the case later in life. This is a strong confirmation of Dr. Black's statements.—*Trans. Ill. State Dental Society.*

